# Making sense of replications

**DOI:** 10.7554/eLife.23383

**Published:** 2017-01-19

**Authors:** Brian A Nosek, Timothy M Errington

**Affiliations:** 1 Center for Open Science, Charlottesville, United States; 2 University of Virginia, Charlottesville, United States

**Keywords:** Reproducibility Project: Cancer Biology, replication, metascience, reproducibility, methodology, open science

## Abstract

The first results from the Reproducibility Project: Cancer Biology suggest that there is scope for improving reproducibility in pre-clinical cancer research.

**DOI:** http://dx.doi.org/10.7554/eLife.23383.001

What is replication? In one sense, the answer is easy. Replication is independently repeating the methodology of a previous study and obtaining the same results. In another sense, the answer is difficult. What does it mean to repeat the methodology? And what qualifies as the "same" results? Now that the first five Replication Studies in the Reproducibility Project: Cancer Biology have been published (see Box 1), it is timely to explore how we might answer these questions. The results of the first set of Replication Studies are mixed, and while it is too early to draw any conclusions, it is clear that assessing reproducibility in cancer biology is going to be as complex as it was in a similar project in psychology (Open Science Collaboration, 2015).

10.7554/eLife.23383.002

Box 1: **The first results from the Reproducibility Project: Cancer Biology**

The first five Replication Studies published are listed below, along with a link to the Open Science Framework, where all the methods, data and analyses associated with the replication are publicly accessible. Each Replication Study also has a figure that shows the original result, the result from the replication, and a meta-analysis that combines these results. The meta-analysis indicates the cumulative evidence across both studies for the size of an effect and the uncertainty of the existing evidence.

Aird F, Kandela I, Mantis C, Reproducibility Project: Cancer Biology. 2017. Replication Study: BET bromodomain inhibition as a therapeutic strategy to target c-Myc. *eLife* **6**:e21253.

Methods, data and analysis available at: https://osf.io/7zqxp/

Horrigan SK, Reproducibility Project: Cancer Biology. 2017a. Replication Study: The CD47-signal regulatory protein alpha (SIRPa) interaction is a therapeutic target for human solid tumors. *eLife* **6**:e18173.

Methods, data and analysis available at: https://osf.io/9pbos/

Horrigan SK, Courville P, Sampey D, Zhou F, Cai S, Reproducibility Project: Cancer Biology. 2017b. Replication Study: Melanoma genome sequencing reveals frequent PREX2 mutations. *eLife* **6**:e21634.

Methods, data and analysis available at: https://osf.io/jvpnw/

Kandela I, Aird F, Reproducibility Project: Cancer Biology. 2017. Replication Study: Discovery and preclinical validation of drug indications using compendia of public gene expression data. *eLife* **6:**e17044.

Methods, data and analysis available at: https://osf.io/hxrmm/

Mantis C, Kandela I, Aird F, Reproducibility Project: Cancer Biology. 2017. Replication Study: Coadministration of a tumor-penetrating peptide enhances the efficacy of cancer drugs. *eLife* **6:**e17584

Methods, data and analysis available at: https://osf.io/xu1g2/

**DOI:** http://dx.doi.org/10.7554/eLife.23383.002

## What does it mean to repeat the methodology?

There is no such thing as exact replication because there are always differences between the original study and the replication. These differences could be obvious (like the date, the location of the experiment, or the experimenters) or they could be more subtle (like small differences in reagents or the execution of experimental protocols). As a consequence, repeating the methodology does not mean an exact replication, but rather the repetition of what is presumed to matter for obtaining the original result.

Direct replication is defined as attempting to reproduce a previously observed result with a procedure that provides no a priori reason to expect a different outcome (Open Science Collaboration, 2015; Schmidt, 2009). In a direct replication, protocols from the original study are followed with different samples of the same or similar materials: as such, a direct replication reflects the current beliefs about what is needed to produce a finding. Conducting a direct replication tests those beliefs empirically. In a conceptual replication, on the other hand, a different methodology (such as a different experimental technique or a different model of a disease) is used to test the same hypothesis: as such, by employing multiple methodologies conceptual replications can provide evidence that enables researchers to converge on an explanation for a finding that is not dependent on any one methodology.

Both direct and conceptual replications are vital to scientific progress. Direct replication can establish that a finding is reproducible. But, on its own, reproducibility does not guarantee validity. For example, the methodology that was repeated could have confounds, or the theoretical explanation for why the finding occurred could simply be wrong. By providing convergent evidence across methodologies, conceptual replication can foster confidence in the explanation for a finding but such evidence, on its own, does not guarantee the reproducibility of any individual piece of evidence. For example, individual findings could have occurred by chance. Together, direct and conceptual replication provides confidence in the reproducibility of a finding and the explanation for the finding.

When a direct replication succeeds, confidence in the original finding increases as does the generalizability of the result (due to the differences between the original and replication methodologies). When a direct replication fails, confidence in the original result decreases, but that does not necessarily mean that the original result was incorrect. It is possible, for example, that differences in the methodologies that were thought to be irrelevant are actually important (Hines et al., 2014). Indeed, a failed replication can lead to a better understanding of a phenomenon if it results in the generation of new hypotheses to explain how the original and replication methodologies produced different results and, critically, leads to follow-up experiments to test these hypotheses (Ebersole et al., 2017).

Together, direct and conceptual replication provides confidence in the reproducibility of a finding and the explanation for the finding.

Discrepancies between the original study and the replication can also be due to error rather than meaningful differences in methodology. For example, a false positive might have been observed by chance in the original study, or a false negative in the replication study: such errors can be caused by low statistical power (Button et al., 2013; Cohen, 1969), or by researchers making design and analysis decisions that increase the likelihood of mistaking noise for signal (Simmons et al., 2011). Errors can also be caused by the improper execution of an experimental technique or by problems with samples and materials (such as the contamination of cell lines; Peterson, 2008). Discrepancies due to error are less interesting than those due to previously unidentified differences in methodology: unfortunately, results rarely provide clear evidence for whether it is one or the other.

The Reproducibility Project: Cancer Biology used a number of strategies to minimize the likelihood that any failure to replicate could be attributed to error (Errington et al., 2014). The teams performing the replications used experimental designs with high statistical power, undertook authentication of key biological materials (such as STR profiling of cell lines), and employed methods to avoid bias (such as randomization). The authors of the original papers were contacted in advance for details of the research methodology that may not have appeared in their paper, and were asked to share any original reagents, protocols and data in order to maximize the quality and fidelity of the replication designs.

There is no straightforward answer to the question "what counts as a successful replication of an original result?"

Moreover, the project is using the Registered Report/Replication Study approach to publish its work and results. The Registered Report details the experimental designs and protocols that will be used for the replications, and experiments cannot begin until this report has been peer reviewed and accepted for publication. The results of the experiments are then published as a Replication Study, irrespective of outcome but subject to peer review to check that the experimental designs and protocols were followed. Finally, all methods, proposed analyses and data are made publicly accessible via the Open Science Framework to maximize transparency and accountability (Nosek et al., 2015). This approach has two main benefits: first, it improves the experimental designs and protocols with expert input prior to performing the experiments; second, by removing the possibility that the results of the experiments will influence the peer review process, it avoids certain biases (such as the bias against negative results, and the possibility that referees will accept results that are favorable to their point of view and reject results that are not; Chambers, 2013; Nosek and Lakens, 2014). Cumulatively, these safeguards maximize rigor, but they do not eliminate the possibility of error.

## What qualifies as the "same" results?

There is no straightforward answer to the question "what counts as a successful replication of an original result?" (Open Science Collaboration, 2015; Valentine et al., 2011). However, asking the following questions will provide some insight: Does the replication produce a statistically significant effect in the same direction as the original? Is the effect size in the replication similar to the effect size in the original? Does the original effect size fall within the confidence or prediction interval of the replication (and vice versa)? Does a meta-analytic combination of results from the original experiment and the replication yield a statistically significant effect? And do the results of the original experiment and the replication appear to be consistent?

Scientific claims gain credibility by accumulating evidence from multiple experiments, and a single study cannot provide conclusive evidence for or against a claim. Equally, a single replication cannot make a definitive statement about the original finding. However, the new evidence provided by a replication can increase or decrease confidence in the reproducibility of the original finding. When a replication "fails" it can spur productive theorizing about the source of that irreproducibility. For example, it could be that the experimental model did not behave as expected (for example, the rate of tumor onset observed in the replication might be higher than the rate observed in the original research in both the control and experimental conditions). In such circumstances, the original hypothesis and finding may not have been evaluated directly because the experimental circumstances necessary to test them did not recur. Alternatively, the model might have behaved as expected, but the experimental intervention did not result in an effect similar to the original study. These two scenarios have different implications for hypothesizing the underlying causes of irreproducibility, and for deciding on the follow-up investigations that are needed to establish reproducibility.

## Conclusion

Replication is a core value of science, and the credibility of scientific claims is based on their reproducibility rather than the authority of their originators. As part of the Reproducibility Project: Cancer Biology the results of all the Replication Studies will be combined to gain insight into the factors that lead to irreproducible results and the opportunities for improving reproducibility (Errington et al., 2014). The results of the first set of Replication Studies suggest that there is a substantial opportunity to improve reproducibility in cancer biology: the challenge facing all of us is to identify how best to achieve this goal.
